# Functional causes of low testosterone predominate in contemporary Australian endocrine referral practice: a two centre experience across 5 years

**DOI:** 10.1007/s12020-026-04722-4

**Published:** 2026-08-08

**Authors:** Aongus O’Brolchain, Kacie McAndrew, William Newman, Dylan Young, Dian Van Zyl, Randunu Dissanyake, Elahe Ebrahimi, Katherine Griffin

**Affiliations:** 1 Griffith University, Southport, QLD Australia; 2 Gold Coast Hospital and Health Service, Southport, QLD Australia

**Keywords:** Testosterone, Andrology, Hypogonadism

## Abstract

**Background:**

The epidemiology and outpatient management of low testosterone in Australian tertiary care remain poorly characterised. Rising testosterone prescription rates and evolving guidelines underscore the need to delineate contemporary referral patterns, diagnostic adherence, and therapeutic practice.

**Methods:**

A retrospective review was undertaken of all new male patients assessed for low testosterone levels at Gold Coast University and Robina Hospitals between 2020 and 2024. Demographic, biochemical, and management data were extracted from the integrated electronic medical record. Continuous variables were analysed using the Kruskal–Wallis test, categorical variables using χ² or Fisher’s exact test, and binary logistic regression identified predictors of testosterone replacement therapy (TRT) initiation and pituitary imaging.

**Results:**

Among 294 consecutive referrals, functional low testosterone (43%) exceeded all pathological diagnostic categories combined individually. Obesity affected 65% of patients and obstructive sleep apnoea 28%, the latter highest in functional low testosterone (48%; *p* < 0.001). Two-thirds of referrals included two early-morning testosterone samples, pituitary imaging was performed in one-quarter of patients, with clinically actionable abnormalities identified in 4% of imaged men. TRT was prescribed in 25% of men, less often in functional than pathological forms (23% vs. 45%; OR 0.37, 95% CI 0.22–0.62; *p* < 0.001). Most patients were discharged after a median of three visits and six months’ follow-up. Functional low testosterone was associated with a high burden of cardiometabolic and psychological comorbidity. Across the entire cohort, 31% reported previous androgen exposure.

**Conclusions:**

Most men referred with low testosterone did not have structural hypothalamic–pituitary–testicular axis disease, supporting a clinical approach prioritising identification of metabolic and reversible contributors before testosterone therapy. Diagnostic completeness and adherence to guideline-recommended imaging vary, but testosterone prescribing generally aligns with current Endocrine Society of Australia (ESA) and Pharmaceutical Benefits Scheme (PBS) restrictions. These findings support the establishment of a dedicated andrology service integrating metabolic, reproductive, and psychological care to optimise assessment and outcomes in men with androgen deficiency.

## Introduction

Hypogonadism is classically recognised in primary testicular disorders, either inherited or acquired (primary hypogonadism) or as a result of pathology in the hypothalamic-pituitary-testicular (HPT) axis (secondary hypogonadism [1, 2]. Pathological androgen deficiency affects approximately 0.5% of men [3]. Klinefelter syndrome remains the most common genetic cause of hypogonadism, affecting 1 in 450–700 men, though it is frequently under-diagnosed due to subtle clinical signs [4]. Pituitary tumors and/or their treatment, genetic disorders and head trauma are examples of aetiologies of secondary hypogonadism.

Historically, the term ‘functional hypogonadism ’ has been used to define the co-existence of androgen-deficiency-like features and low serum testosterone in the absence of intrinsic structural HPT axis pathology [5]. Functional hypogonadism typically occurs in men with comorbidities such as overweight, obesity, and diabetes [6]. It may also be seen with chronic opioid or glucocorticoid exposure. However, use of this term is controversial, as it may imply a pathological state requiring treatment. Recently, new terminology has been suggested when the cause of functional low testosterone is obesity: ‘pseudohypogonadism of obesity’ [7]. Accordingly, careful clinical assessment is required to distinguish pathological hypogonadism from non-gonadal influences on testosterone concentrations. ‘Functional low testosterone’ is potentially reversible and affects up to 12–15% of community-dwelling adult males [8].

Obesity and metabolic comorbidities are major reversible contributors to low testosterone. In 2022, 71% of Australian men were overweight or obese, rising to 81% among those aged 65–74 years [9]. Men with a body mass index (BMI) of 35–40 kg/m^2^ can have up to half the total testosterone when compared to age-matched peers with a normal BMI [10, 11]. A bidirectional Mendelian randomization analysis including over seven thousand Caucasian men predicted that a reduction in BMI from 30 to 25 kg/m^2^ would result in a 13% increase in circulating testosterone [12]. Similarly, another study looking at dietary interventions and bariatric surgery found for each 1 kg of weight lost, between baseline and 36 months, total testosterone increased by 0.6% [13].

Exogenous testosterone and synthetic androgen exposure suppress the hypothalamic–pituitary–gonadal axis, reduce intratesticular testosterone concentrations, impair spermatogenesis, and may result in infertility and testicular atrophy. Testosterone prescribing in Australia has increased dramatically over the past two decades without any new therapeutic indications [1]. Despite stable prevalence estimates of pathological hypogonadism, testosterone prescriptions rose more than three-fold between 2000 and 2015, driven primarily by primary care prescribers [2, 3]. Pharmaceutical Benefits Scheme (PBS) expenditure increased from less than $20,000 per 100,000 individuals in 2000 to over $110,000 in 2018 [14]. In 2015, the PBS introduced stricter restrictions, requiring specialist endorsement and evidence of pituitary or testicular pathology for the first prescription. Concurrently, the Endocrine Society of Australia (ESA) issued a position statement outlining the indications for testosterone use [1]. In the absence of true pathological hypogonadism, testosterone replacement therapy (TRT) remains controversial [15].

The increasing prevalence of obesity, mirrored by increases in testosterone prescriptions, portend an increased demand on primary care and endocrinology services in Australia which underscores the need for increased training and further research to guide current and future clinical practice.

We hypothesised that contemporary endocrine referrals for low testosterone would predominantly comprise men with functional low testosterone rather than pathological disorders of the HPT axis, and that these men would demonstrate a higher burden of metabolic comorbidity and lower rates of testosterone prescribing.

## Methods

All new male patients referred for assessment of possible hypogonadism or androgen deficiency between 1 January 2020 and 31 December 2024 were eligible for inclusion. All consecutive referrals were included irrespective of attendance status in order to characterise referral practice. Referrals were accepted from general practitioners, hospital specialists, and internal departments when the referral explicitly mentioned low testosterone, symptoms of androgen deficiency, or infertility where biochemical hypogonadism was suspected. Men referred exclusively for fertility evaluation with normal androgen profiles were excluded. Duplicate referrals across Robina and Gold Coast University Hospitals were identified and de-duplicated.

Demographic variables included age and body mass index (BMI). Clinical variables included smoking history, alcohol intake, obesity, obstructive sleep apnoea (OSA), type 2 diabetes (T2DM), atherosclerotic cardiovascular disease (ASCVD), chronic kidney disease (CKD), previous testicular insult (trauma, mumps orchitis, malignancy, chemotherapy, torsion, cryptorchidism or hydrocele), and prior exposure to testosterone, androgens, glucocorticoids and opioids. Symptoms of androgen deficiency (including erectile dysfunction, low libido, fatigue, depression and gynaecomastia) and medication use (antidepressants, glucocorticoids and opioids) were also recorded.

### Referral and assessment data

Information was extracted including referring clinician type and referral completeness (inclusion of two early-morning testosterone levels and gonadotrophins).

Biochemical variables relevant to diagnostic classification and management included first and second morning total testosterone concentrations, sex hormone-binding globulin (SHBG), luteinising hormone (LH), follicle-stimulating hormone (FSH), and selected investigations performed at clinician discretion (e.g. prolactin and iron studies where clinically indicated).Documentation of physical examination findings (testicular examination and volume in millilitres, presence or absence of gynaecomastia) were recorded.

Ordering and results of pituitary imaging (either computerised tomography (CT) or magnetic resonance imaging (MRI) in patients with hypogonadism with inappropriately normal or low LH was assessed in line with Endocrine Society guidelines [16].

### Diagnosis and management

Diagnostic classification was assigned based on treating clinician diagnosis from the medical record. Where documentation was ambiguous or incomplete, biochemical parameters were used to confirm classification following specialist chart review.

*Primary hypogonadism* was defined as low serum testosterone with elevated LH and/or FSH or known testicular pathology.

*Secondary hypogonadism* comprised structural hypothalamic–pituitary disease associated with low testosterone and low or inappropriately normal gonadotrophins.

*Functional low testosterone* was defined as low morning testosterone with low or inappropriately normal gonadotrophins in the absence of recognised structural hypothalamic–pituitary or testicular disease. Diagnostic classification was based primarily on treating clinician assessment within routine endocrine practice, with biochemical thresholds used descriptively rather than as sole diagnostic criteria. Drug-associated suppression due to opioids or glucocorticoids was classified within functional low testosterone where no structural HPT-axis pathology was identified.

*Exogenous androgen-related* hypogonadism referred to current or prior androgen exposure with subsequent suppression of the HPT axis.

Men without confirmed pathological hypogonadism, including eugonadal presentations, indeterminate biochemical findings, and miscellaneous syndromic or idiopathic causes, were combined into a single “other” category for statistical analysis due to small subgroup sizes.

Treatment data included use of testosterone therapy, clomiphene, human chorionic gonadotrophin (hCG), or other agents. Fertility counselling/referral, discharge status, and ongoing follow-up were also recorded.

Serum total testosterone concentrations were obtained from routine pathology reports accompanying referrals or performed within the public hospital system. As this retrospective audit reflected routine clinical practice, assays were performed by multiple accredited public and private pathology providers using their standard analytical platforms. Information regarding assay methodology (including whether liquid chromatography–tandem mass spectrometry [LC-MS/MS] or immunoassay was used) was not consistently available from historical referral documentation and therefore could not be standardised retrospectively.

### Statistical analysis

Continuous variables were assessed for normality using the Shapiro–Wilk test and expressed as mean ± SD or median (IQR) as appropriate. Between-group comparisons were performed using the Kruskal–Wallis test for continuous data and χ² or Fisher’s exact test for categorical data. Where the Kruskal–Wallis test was significant, post-hoc pairwise comparisons versus the eugonadal group were performed using Dunn’s test with Holm adjustment for multiple comparisons. Multivariable logistic regression examined the association between age, BMI, total testosterone concentration, LH concentration and TRT initiation or pituitary imaging. A two-tailed *p* < 0.05 was considered significant. Analyses were conducted using Stata 18 (StataCorp, College Station, TX, USA).

This study was approved as a service evaluation by the Gold Coast Hospital and Health Service Human Research Ethics Committee ((LNR) HREC/2025/QGC/121038) and conducted in accordance with institutional governance requirements.

## Results

### Diagnostic distribution and baseline characteristics

**Fig. 1:**
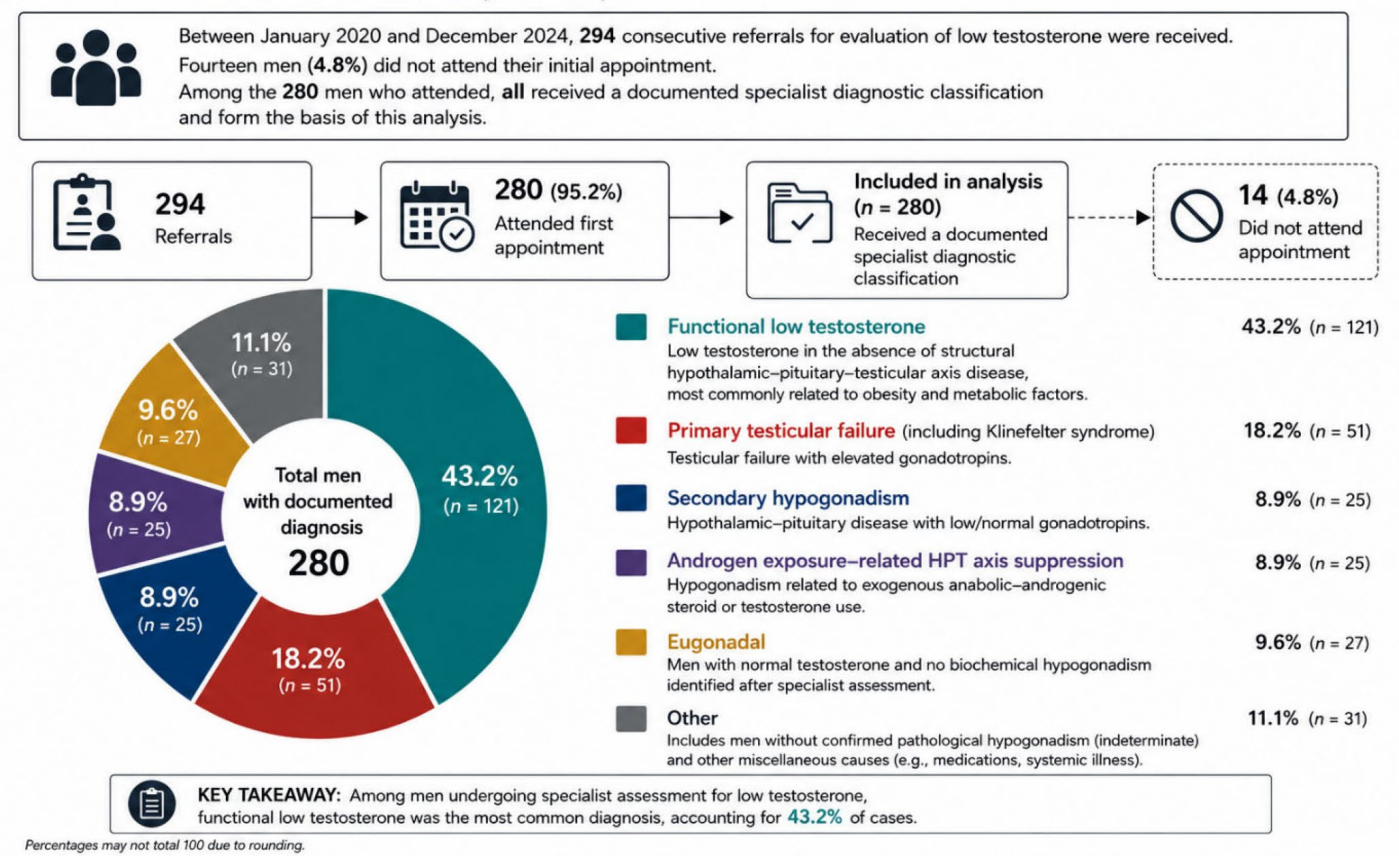
Diagnostic categories among men undergoing specialist assessment for low testosterone (*n*=280)

**Fig. 2:**
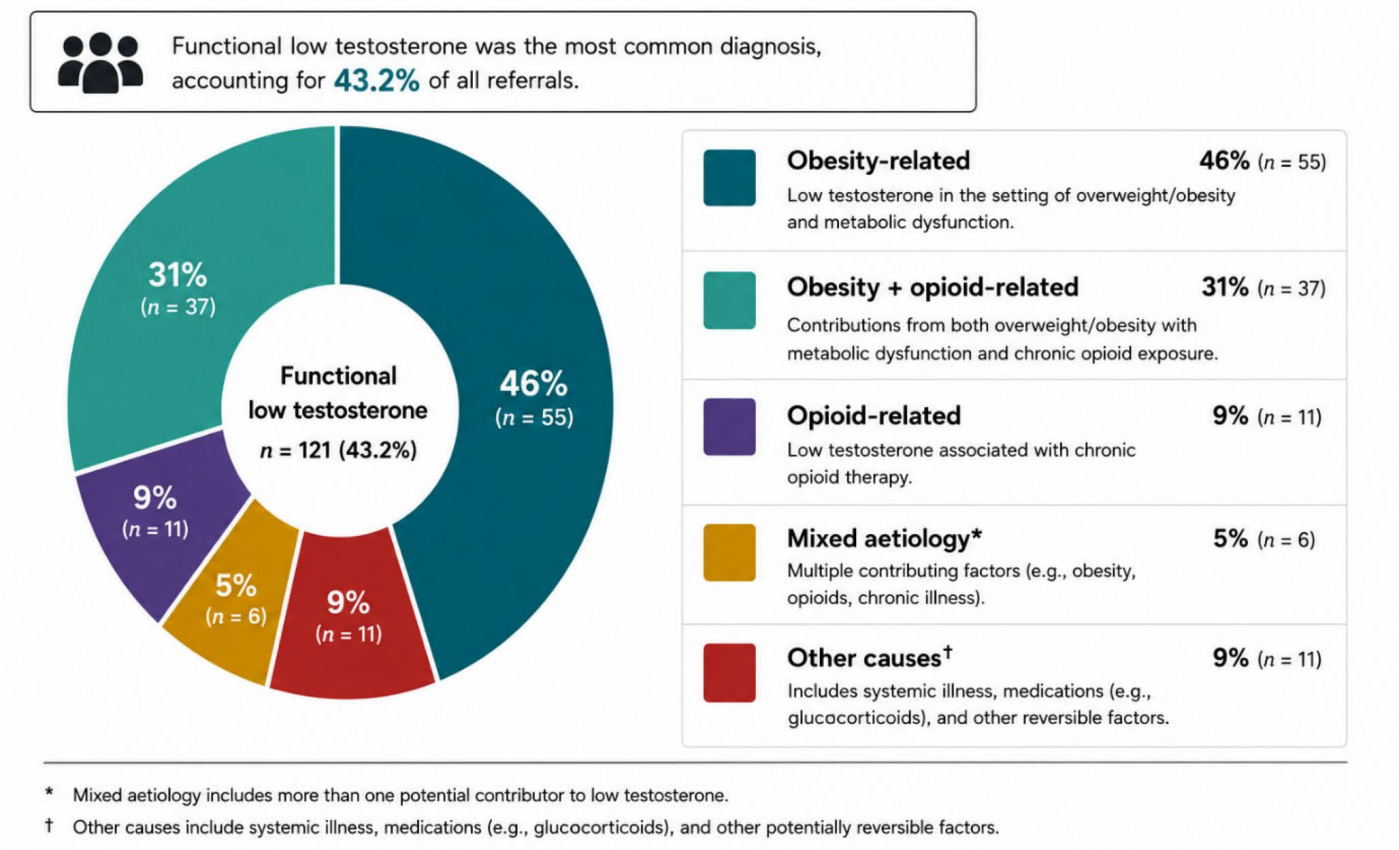
Subtypes of functional low testosterone (n=121)

Between January 2020 and December 2024, 294 consecutive referrals for evaluation of low testosterone were received. Fourteen men (4.8%) did not attend their initial appointment. Diagnostic analyses therefore included the 280 men who completed specialist assessment and received a final diagnostic classification (Fig. 1). Functional low testosterone was the most common diagnosis, accounting for 121 men (43.2%), followed by primary testicular failure (51; 18.2%), androgen exposure–related HPT axis suppression (25; 8.9%), secondary hypogonadism (25; 8.9%), eugonadal presentations (27; 9.6%) and other diagnoses (31; 11.1%). Obesity alone accounted for almost half of functional low testosterone, while obesity combined with chronic opioid exposure accounted for a further one-third (Fig. 2). Together, obesity alone or in combination with chronic opioid use accounted for over three quarters of functional low testosterone.

Significant differences between diagnostic groups were observed for BMI and several biochemical parameters (Table 1). Obesity was present in 65.2% of the cohort and was most prevalent among men with functional low testosterone (79.8%; *p* < 0.001). Obstructive sleep apnoea affected 28.6% overall and almost half of men with functional low testosterone (48.8%; *p* < 0.001). Type 2 diabetes was also more common in functional low testosterone than in several other diagnostic groups (23.1%; *p* = 0.03). No significant differences were observed for ASCVD or chronic kidney disease (Table 2).

Among the 80 men with documented obstructive sleep apnoea (based on self-report or sleep study where available), 46% were prescribed or using continuous positive airway pressure (CPAP), with no significant difference in adherence between obesity-related and non-obesity-related functional low testosterone (*p* = 0.33).

**Table 1:** Demographic and biochemical characteristics according to diagnostic classification

Variable	Functional low testosterone	Secondary hypogonadism	Primary testicular failure	Androgen exposure	Eugonadal	Other	Total	*P* value
*n*	121	25	51	25	27	31	280	-
Age (years)	46 [37–57]	40 [22–51]	51 [34–67.5]	47 [34–55]	52 [37.5–60.5]	42 [28–53.5]	46.5 [34–58]	0.057
BMI (kg/m²)	34.2 [30–38.9]*	29 [26.7–31.7]	30.1 [27.5–34]	30 [28–32]	27.4 [23.2–31.3]	28 [23.5–32.5]	31.4 [27.7–36.9]	< 0.001
Right testicular volume (mL)	17.4 [15–20]	13 [6–15]	10.7 [6.5–15]	18 [9–20]	20 [17–20]	13.5 [10–16.2]	15 [10–20]	0.05
Left testicular volume (mL)	19 [15–25]	11 [6.5–15]	10 [7–18.5]	19 [16.2–20]	20 [17–24]	10 [9.7–20]	15 [10–20]	0.08
First testosterone (nmol/L)	5.9 [3.8–8]*	5.5 [3.4–7.5]*	5.4 [2.8–8.4]*	5.8 [3.8–15.3]	9 [6.1–11]	5.9 [4.1–8]*	6 [3.7–8.9]	0.027
Second testosterone (nmol/L)	6.2 [4.5–8.1]*	7 [3.2–9.9]*	5.1 [3.1–7.3]*	7 [5.5–22.6]	11.7 [8.6–14]	7 [4.8–8.9]*	6.9 [4.2–10]	< 0.001
SHBG (nmol/L)	18 [13.8–29]	20 [14.8–24]	30 [19–43]	18.5 [12.2–22.9]	25.5 [19.2–35.8]	24 [15.5–38.2]	21 [14–33]	0.004
LH (IU/L)	3.0 [2.0–4.0]	3.0 [1.0–4.2]	14 [5.2–20.5]*	1.0 [0.5–3.0]	3.2 [2.0–4.9]	3.0 [2.0–5.5]	3.0 [2.0–6.0]	< 0.001
FSH (IU/L)	4.0 [2.0–6.5]	4.5 [2.1–7.2]	26 [11–45]*	2.0 [1.5–3.5]*	6.0 [4.0–9.0]	7.0 [5.0–10]	5.2 [2.2–10]	< 0.001
Haemoglobin (g/L)	150 [143–158]	143 [135–160]	141 [135.5–153]	155.5 [142–164.2]	152 [141–156]	142 [139–150]	148 [140–157]	0.014
Haematocrit	0.46 [0.43–0.48]	0.44 [0.43–0.48]	0.43 [0.40–0.47]	0.47 [0.43–0.49]	0.46 [0.42–0.47]	0.44 [0.41–0.45]	0.45 [0.43–0.48]	0.042

Values are median [IQR]. Analyses include attended patients with an assigned diagnosis (n=280). Primary testicular failure includes Klinefelter syndrome. Secondary hypogonadism includes pituitary/hypopituitary, idiopathic hypogonadotropic hypogonadism, developmental delay, hyperprolactinaemia and genetic cause NOS. P values derived from Kruskal-Wallis tests. . * Significantly different from the eugonadal group on Dunn's post-hoc multiple-comparison test with Holm adjustment (adjusted p < 0.05); SHBG = sex hormone-binding globulin; LH = luteinising hormone; FSH = follicle-stimulating hormone.

### Comorbidities, medication exposure and presenting symptoms

Exposure to exogenous androgens occurred in 31% of men. Antidepressant exposure was significantly more common in functional low testosterone, consistent with the higher prevalence of depressive symptoms observed in this group. Erectile dysfunction, fatigue, and low libido were common across all diagnostic categories, highlighting substantial clinical overlap between functional and pathological hypogonadism. (Table 2).

### Management and diagnostic assessment

TRT was prescribed to 73 patients (25%), with 32 already receiving treatment. It was less common in functional low testosterone than pathological hypogonadism (23% vs. 45%, *p* < 0.001), corresponding to a threefold lower likelihood of use (OR 0.37, 95% CI 0.22–0.62). New initiation (17% vs. 30%, *p* = 0.011) and pre-existing therapy (6% vs. 15%, *p* = 0.017) were similarly lower. Documented complications occurred in 22%, most commonly self-cessation, polycythaemia, or mood disturbance; rates did not differ by indication (*p* = 0.48).

**Table 2:** Clinical characteristics according to diagnostic classification

Variable	Functional low testosterone	Secondary hypogonadism	Primary testicular failure	Androgen exposure	Eugonadal	Other	Total	*P* value
*n*	121	25	51	25	27	31	280	-
**Comorbidities**								
Obesity	95 (79.8%)	10 (47.6%)	27 (58.7%)	13 (61.9%)	6 (31.6%)	12 (50.0%)	163 (65.2%)	< 0.001
Obstructive sleep apnoea	59 (48.8%)	3 (12.0%)	10 (19.6%)	2 (8.0%)	2 (7.4%)	4 (12.9%)	80 (28.6%)	< 0.001
Type 2 diabetes	28 (23.1%)	1 (4.0%)	12 (23.5%)	4 (16.0%)	1 (3.7%)	3 (9.7%)	49 (17.5%)	0.03
ASCVD	16 (13.2%)	1 (4.2%)	7 (13.7%)	5 (20.0%)	1 (3.7%)	0 (0.0%)	30 (10.8%)	0.062
Chronic kidney disease	7 (5.8%)	1 (4.0%)	3 (5.9%)	2 (8.0%)	0 (0.0%)	0 (0.0%)	13 (4.7%)	0.588
Any testicular insult	7 (5.9%)	1 (4.2%)	16 (32.7%)	3 (12.5%)	2 (7.4%)	3 (9.7%)	32 (11.7%)	< 0.001
**Medication exposure**								
Previous testosterone/androgen exposure	22 (18.8%)	8 (32.0%)	19 (38.0%)	24 (96.0%)	4 (16.0%)	6 (21.4%)	83 (30.7%)	< 0.001
Duration of testosterone/androgen exposure (years), median [IQR]	3.5 [1–8]	3 [1.8–5]	2.5 [1–6.5]	7.5 [5–11.2]	1 [1–3.2]	4 [1.5–4]	4.5 [1–8]	0.010
Antidepressant use	42 (35.3%)	8 (33.3%)	7 (13.7%)	3 (12.5%)	6 (22.2%)	4 (13.3%)	70 (25.5%)	0.010
Opioid use	26 (22.0%)	2 (8.0%)	3 (5.9%)	2 (8.0%)	2 (7.4%)	3 (10.0%)	38 (13.8%)	0.052
Glucocorticoid use	8 (6.7%)	2 (8.0%)	0 (0.0%)	1 (4.0%)	2 (7.4%)	0 (0.0%)	13 (4.7%)	0.187
**Symptoms and signs**								
Erectile dysfunction	53 (48.6%)	6 (28.6%)	16 (33.3%)	9 (37.5%)	9 (36.0%)	12 (40.0%)	105 (40.9%)	0.355
Low libido	77 (70.6%)	12 (52.2%)	27 (57.4%)	12 (50.0%)	12 (46.2%)	20 (66.7%)	160 (61.8%)	0.098
Fatigue	85 (75.9%)	14 (56.0%)	34 (70.8%)	14 (58.3%)	18 (69.2%)	19 (63.3%)	184 (69.4%)	0.279
Depression	51 (42.1%)	6 (24.0%)	7 (13.7%)	8 (33.3%)	8 (29.6%)	7 (23.3%)	87 (31.3%)	0.008
Gynaecomastia	23 (20.9%)	6 (27.3%)	6 (12.5%)	2 (8.7%)	3 (11.5%)	5 (16.7%)	45 (17.4%)	0.449

Values are n (%) unless otherwise stated. Denominators vary because of missing data. Categorical p values were derived from Pearson chi-square tests, with Fisher exact tests using Monte Carlo simulation where expected cell counts were small; continuous p value derived from Kruskal-Wallis test. OSA = obstructive sleep apnoea; ASCVD = atherosclerotic cardiovascular disease

### Diagnostic evaluation

Pituitary imaging was performed in approximately one-quarter of patients and was more common in men with severe biochemical hypogonadism and low or inappropriately normal gonadotrophins. Among imaged patients, most abnormalities were incidental microadenomas or empty sella variants, while clinically actionable lesions were uncommon (4%). Scrotal ultrasound or examination was recorded in 47%, both in 10%. Men with elevated LH (≥ 12 IU/L) were less likely to have a testicular examination documented (OR 0.49, 95% CI 0.28–0.86). Gynaecomastia was recorded in 17.4% of patients.

A weight-loss trial was documented in 63% of obesity-related functional low testosterone cases, and 30% received non-testosterone interventions (10 patients (3.4%) were receiving or were commenced on GLP-1 receptor agonists). Use of such therapies did not differ between obesity-related and other functional causes (*p* = 0.99).

Two separate morning testosterone levels were included in 66% of referrals, early-morning sampling (0600–1000 h) in 64%, and LH/FSH in 65%.

## Discussion

This study demonstrates that most men referred for specialist evaluation of low testosterone have functional low testosterone rather than pathological hypothalamic–pituitary–testicular disease, with obesity and metabolic dysfunction representing the dominant associated factors.

As few as 25% of men with Klinefelter’s syndrome receive a diagnosis, underscoring the importance of testicular examination in patients with hypergonadotrophic hypogonadism, with many experts considering this to be a mandatory assessment in the work-up of apparent androgen deficiency and male infertility [7, 17]. Although guidelines recommend that elevated LH levels prompt evaluation for primary testicular failure, this study identified a paradoxical trend: men with raised LH were less likely to have a documented testicular examination. These findings highlight incomplete adherence to guideline-recommended assessment. Measurement of gonadotrophins and genital examination are fundamental components of evaluating men with low testosterone, analogous to measuring both TSH and free thyroxine when assessing thyroid dysfunction. Possible explanations include under-documentation and a referral bias where an established diagnosis may obviate the need for re-examination. This underscores the need for structured templates or electronic prompts to ensure examination documentation in endocrine referrals.

Endocrine Society guidelines advise pituitary imaging (computerised tomography or MRI) when biochemical findings suggest central hypogonadism, where the likelihood of structural hypothalamic–pituitary disease is highest [16]. Pituitary imaging was performed in approximately one-quarter of patients, reflecting appropriate clinical discretion given heterogeneous presentations [1, 5, 16]. These findings suggest that targeted imaging based on biochemical severity remains appropriate but could be streamlined using clear clinical triggers.

The Australian PBS and Endocrine Society of Australia criteria require documented pathological hypogonadism for subsidised TRT [14]. Not all men with pathological hypogonadism commenced testosterone therapy during the study period. This reflected routine clinical practice, whereby some men wished to preserve fertility and were managed with alternative therapies (e.g. clomiphene citrate or human chorionic gonadotrophin), some were undergoing further diagnostic evaluation before treatment, and others declined treatment or remained under observation pending repeat assessment. A minority of men with functional low testosterone received TRT despite the absence of recognised structural HPT axis disease. Owing to the retrospective design, it was not possible to determine whether these prescriptions were privately funded, initiated before specialist review, or fulfilled PBS eligibility criteria in every case.

The high prevalence of obesity (79%) and OSA (48%) among men with FLT supports the view that, in contemporary Australian practice, this condition largely reflects reversible metabolic and sleep-related suppression of the HPT axis. In a meta-analysis of diet and exercise interventions, approximately 10% body-weight reduction was associated with an increase in total testosterone of about 2.9 nmol/L [18]. In surgical cohorts, weight loss averaging ~ 32% of baseline body weight produced increases of 8.7–9.0 nmol/L in total testosterone [19].

These observations - that aggressive lifestyle modification with weight loss results in proportionate increases in serum testosterone - have led to interest in incretin mimetic therapy (IMT) in the management of functional low testosterone [20]. Emerging data suggest that weight reduction via IMT produces greater increases in serum testosterone and reductions in oestradiol compared with TRT alone [21]. Though most patients in this study were appropriately advised to pursue weight reduction, 37% lacked a documented trial of weight loss, highlighting an opportunity to strengthen multidisciplinary engagement.

OSA was highly prevalent among men with functional low testosterone, although this association is likely mediated predominantly through shared metabolic factors including obesity and insulin resistance rather than direct suppression of the HPT axis. Although some studies have demonstrated an inverse relationship between OSA severity and testosterone levels [11], The effect on serum testosterone with treatment of OSA with continuous positive airway pressure (CPAP) is controversial [22]. TRT is generally considered to be contraindicated in severe, untreated OSA [16], but the evidence is weak [23] and current ESA guidelines do not consider this an absolute contraindication [1]. Non-compliance with CPAP may dissuade clinicians from commencing TRT in obese patients, although most studies show minimal effect of OSA treatment on gonadotrophs or serum testosterone [24]. Other conditions that can cause lower serum testosterone in men include type 2 diabetes [25], chronic liver disease [26], haemochromatosis [27] and chronic kidney disease [28].

The predominance of functional low testosterone underscores the need to integrate metabolic and psychological care within andrology pathways rather than empirical testosterone prescribing. This aligns with current international and Australian guidelines that prioritise correction of underlying drivers: obesity, insulin resistance and opioid use.

The concept of “functional hypogonadism” remains debated. While widely used to describe potentially reversible suppression of the HPT axis due to obesity, systemic illness, or medications, it risks conflating a biochemical state with a pathological diagnosis. In many cases, low testosterone reflects non-gonadal factors such as reduced SHBG or chronic disease burden and does not represent intrinsic androgen deficiency. Our findings support the view that, in a tertiary referral population, most men with low testosterone do not have structural HPT axis disease, and that clinical assessment should prioritise identification of underlying comorbidities rather than empirical testosterone prescribing.

This study has several limitations. Its retrospective design introduced potential ascertainment and documentation bias, particularly for examination findings and symptom assessment. Diagnostic classification reflected the assessment of endocrinologists within routine clinical practice rather than predefined research criteria. Consequently, some men classified as having pathological disease may have had functional low testosterone, and some degree of diagnostic overlap or misclassification is inevitable in a retrospective study. Nevertheless, diagnoses were based on the integration of clinical history, examination findings, biochemical assessment and imaging, reflecting real-world specialist practice. Missing biochemical data reflected selective clinician ordering rather than protocolized assessment. Hyperprolactinaemia cases are likely to be underrepresented in this cohort, as in this tertiary centre, micro and macroprolactinomas are assessed and managed in a dedicated pituitary clinic. Testosterone measurements were derived from multiple accredited laboratories, and assay methodology was not consistently available from referral documentation. Consequently, it was not possible to determine whether measurements were performed using LC-MS/MS, the preferred reference method, or immunoassay in every case. However, this reflects real-world Australian referral practice and enhances the external validity of the findings.

Longitudinal follow-up of men with functional low testosterone was beyond the scope of the present audit but represents an important future direction, particularly to evaluate the effectiveness of structured weight management, treatment of sleep-disordered breathing, and optimisation of metabolic comorbidity on recovery of endogenous testosterone concentrations.

To our knowledge, this represents the first comprehensive review of low testosterone referral management within an integrated Australian health service, demonstrating real-world guideline adherence and highlighting targets for service redesign.

## Conclusion

Functional causes of low testosterone predominated among men referred for evaluation of low testosterone in this Australian tertiary cohort and was strongly associated with obesity, OSA, and psychological comorbidity. Most patients did not have structural hypothalamic–pituitary–testicular disease, supporting a multidisciplinary approach prioritizing metabolic, psychological, and lifestyle interventions alongside selective hormonal therapy where appropriate.

## Data Availability

The datasets generated and analysed during the current study are available from the corresponding author on reasonable request and subject to approval by Gold Coast Health Human Research Ethics and Governance processes.
